# Hepatitis B Immunisation amongst doctors and laboratory personnel in KwaZulu-Natal, South Africa

**DOI:** 10.4102/phcfm.v5i1.452

**Published:** 2013-04-15

**Authors:** Feroza Y. Khan, Andrew J. Ross

**Affiliations:** 1 Department of Family Medicine, University of KwaZulu-Natal, South Africa

## Abstract

**Background:**

Hepatitis B virus (HBV) infection is the most easily transmitted blood-borne pathogen and is an occupational hazard for health care workers (HCWs). Despite the fact that infection is preventable through vaccination and post-exposure immunoglobulin therapy, many HCWs are unaware of the risks of HBV infection and of appropriate preventative measures. This study is unique in the South African setting as it focuses on the exposure, attitude and knowledge of doctors to HBV infection.

**Method:**

This was an observational descriptive study. Records of the HBV immune status of all doctors who reported an occupational injury (OI) to the occupational health clinic between June 2010 and May 2011 were reviewed. A structured questionnaire was then distributed to all laboratory personnel and senior doctors employed at the hospital.

**Results:**

Of the 67 doctors who reported an OI, 39% (26 out of 67) had no HBV immunity and only 19% (5 out of 26) had received Hepatitis B immunoglobulin. Of the 78 doctors who completed the questionnaire, 65% (51 out of 78) reported at least one OI during their career. Fifty-six percent of the respondents were unaware of their HBV immune status and only 31% had received a booster within the previous 5 years.

**Conclusion:**

Poor compliance of HCWs to HBV vaccination and post-exposure prophylaxis is a concern. In-service training is needed to inform staff of the efficacy of HBV vaccination and immunoglobulin therapy.

## Abstrait

**L'immunisation contre l'hépatite B chez les médecins et le personnel de laboratoire dans le KwaZulu-Natal, en Afrique du Sud**

**Contexte:**

L'infection par le virus de l'hépatite B (VHB) est l'agent pathogène transmis par le sang qui se transmet le plus facile. Ceci constitue un risque du métier pour les professionnels de la santé (PS). Malgré le fait que l'infection puisse être évitée par la vaccination et l'administration post-expositionnelle d'immunoglobulines, beaucoup de PS n'ont pas conscience des risques d'infection par le VHB et des mesures préventives appropriées. Cette étude est unique dans le contexte sud-africain car elle se concentre sur l'exposition, l'attitude et la connaissance par les médecins de l'infection par le VHB.

**Méthodologie:**

Il s'agit d'une étude descriptive observationnelle. Les historiques relatifs au statut immunitaire vis-à-vis du VHB de tous les médecins ayant signalé un accident du travail (AT) à l’établissement de santé qui les employait entre juin 2010 et mai 2011 ont été examinés. Un questionnaire structuré a ensuite été distribué à tout le personnel de laboratoire et aux chefs de service employés par l'hôpital.

**Résultats:**

Parmi les soixante-sept médecins qui ont signalé un AT, 39% (26 de 67) n’étaient pas immunisés contre le virus de l'hépatite B et seulement 19% (5 de 26) avaient reçu des immunoglobulines de l'hépatite B. Parmi les 78 médecins qui ont répondu au questionnaire, 65% (51 de 78) ont signalé au moins un AT au cours de leur carrière. Cinquante-six pour cent des personnes interrogées ne connaissaient pas leur statut immunitaire vis-à-vis du VHB et seulement 31% avaient reçu un vaccin de rappel au cours des cinq dernières années.

**Conclusion:**

La mauvaise observance par le personnel de santé de la vaccination contre le VHB et la prophylaxie post-expositionnelle est inquiétante. Une formation interne est nécessaire pour informer le personnel de l'efficacité de la vaccination contre le VHB et de l'administration d'immunoglobulines.

## Introduction

### Key focus

Hepatitis B virus (HBV) infection is a recognised occupational hazard for health care workers (HCW) who are in contact with infected body fluids and sharp instruments.^1^ The *Occupational Health & Safety Act of 1993* was promulgated to protect HCWs against this preventable disease. It stipulates that all HCWs at risk must be immunised against HBV^2^ and makes the use of appropriate personal protective measures (gloves, goggles, safe disposal of needles, etc.) compulsory. Personal observation at the occupational health clinic (OHC) at the combination hospital (regional and district) where the researcher is based suggested that the Department of Health's (DOH) obligation in terms of the Act^2, 3^ to ensure that all HCWs are appropriately protected against HBV infection was not being met, especially amongst senior doctors and laboratory personnel.

The aim of the study was to investigate the validity of these observations and to make recommendations, based on the findings, to the hospital management to enhance HCWs’ protection.

#### Background

Hepatitis B virus (HBV) infection is a recognised occupational hazard for health care workers (HCWs) who are in contact with blood, body fluids and sharp instruments. Infection may result after exposure by means of needle sticks, cuts from other contaminated sharp instruments or mucosal contact.^4^ HBV is a hepatotropic virus that may cause a self-limiting illness or progress to a more chronic disease, increasing the risk of liver cirrhosis or hepatocellular carcinoma.^5^.

According to South African legislation,^2^ every employer must have a system in place to ensure that all staff are immunised against HBV infection and that exposures to HBV and other blood-borne pathogens are reported and appropriately managed. HBV infection in the workplace is a preventable disease provided all at risk personnel are immunised with the HBV vaccine and develop immunity. The development of such immunity can be determined by testing for hepatitis B surface antibodies (HepBsAb).^6^ The KwaZulu-Natal DOH has made provision for hepatitis immunisation to be made available through OHCs to all staff members who have not previously been immunised and for those who need booster immunisation.^3^

In the occupational health setting HBV is the most easily transmitted blood-borne pathogen, followed by Hepatitis C virus, and then HIV.^7^ The risk of a HCW acquiring HBV infection after a needle stick injury is one hundred times greater than the risk of acquiring HIV infection. The non-immune HCW has a 27% to 37% risk of contracting hepatitis B if the source patient is HBeAg negative. The risk increases to 62% if the source patient is HBeAg positive.^8^

The HBV vaccine is safe and effective.^3^ Pre-exposure vaccination is freely available in all government institutions, including universities and hospitals. It is best administered when the HCW is undergoing training. Immunisation involves three intramuscular injections followed by post-vaccination testing one to two months after the last dose to assess immunity.^3^ A HBV surface antibody titre of >10muIU per mL indicates the presence of immunity.^3^

The Immunisation Action Coalition recommends that HCWs with a normal immune status who demonstrate an adequate hepatitis B surface antibody titre response of at least 10mIU per mL following their vaccination series do not need a booster or periodic HepBsAb testing. This recommendation is based on the fact that although HepBsAb titre levels decline with time, the immune memory in the form of the anamnestic anti-HBs response remains intact indefinitely. Therefore the HCW with HepBsAb levels <10mIUper mL remains protected.^9^ However, the South African National Department of Health (DOH) guidelines advocate administration of a HBV booster every five years with no need for further immunity testing, unless the HCW has sustained a percutaneous or mucosal injury.^10^ This recommendation is based on the high prevalence of HIV infection in South Africa and the assumption that the immune status of HCWs may be challenged by HIV.^10^

Post-exposure prophylaxis (PEP) with HBV immunoglobulin provides substantial protection following exposure to blood and blood products for HCWs who have no immunity against HBV infection.^11^

The current DOH HBV PEP recommendations following exposure to blood or blood products (see Table 1).^12^

**TABLE 1:** Department of Health recommendations for Hepatitis B virus Post-exposure prophylaxis following exposure 12.

Status of health care worker	Status of patient	Action to be taken following exposure
Vaccinated HBsAb > 10 mlIU per mL	Hep B infected	No further action needed
Vaccinated HBsAb < 10 mlIU per mL	Hep B infected	HBIG within 24 hours + booster vaccination
Uncertain vaccination or no previous vaccination	Hep B infected	HBIG within 24 hours followed by vaccination series
Uncertain vaccination or no previous vaccination	Hep B un infected	Hepatitis B vaccination series

HBIG, Hepatitis B immunoglobulin.

The importance of personal protection cannot be over-emphasised as the risk of HBV infection can be substantially reduced by adherence to strict infection control practices and the appropriate use of barrier precautions to prevent skin and mucous membrane exposure when handling infected blood or other body fluids.^12^

#### Trends

HBV infection is a global health problem with approximately 350 million HBV carriers reported worldwide and 500 000 deaths occurring each year. In South Africa acute and chronic HBV infection occurs commonly in the Black population but rarely in other population groups. The prevalence of chronic HBV infection is 5% – 16% in rural Black males, 8% – 9% in urban Black males, 4% – 12% in rural Black females and 2.7% – 4% in urban Black females. Approximately 3 – 4 million South African Blacks are chronically infected with HBV.^6^

It is estimated that 5.9% of HCWs are exposed annually to HBV. This equates to approximately 66 000 HBV infections worldwide.^13^

A number of international studies carried out in Kenya in 2006^14^ and in Albania in 2007^15^ have shown a high prevalence of HBV infection amongst the general population and low vaccination coverage amongst HCWs. These studies are consistent with South African studies.^6^ In 2002 Vardas et al.^16^ showed that only 30.6 percent of HCWs were immune to HBV infection and in 2007 in Bloemfontein De Villiers^8^ showed that in 67.5% of cases the HCWs’ HBV status was unknown following an occupational exposure. There is a paucity of data regarding the true extent of immunity against HBV infection amongst doctors in South Africa in general and in KwaZulu-Natal in particular and this study sought to fill this gap.

#### Objectives

1. To determine the hepatitis B immune status of staff who sustained a sharp injury at a combination hospital in KwaZulu-Natal.
2. To assess the knowledge and attitude of doctors and laboratory personnel with regards to Hepatitis B infection and immunisation.

#### Contribution to field

Occupationally acquired HBV infection is a serious yet preventable health hazard to all at risk employees. Despite the availability of the HBV vaccine and the Hepatitis B immunoglobulin (HBIG), national and international studies have shown that a significant number of HCWs are inadequately immunised against this harmful virus. This study will contribute to the field of knowledge by assessing HCWs’ knowledge of HBV infection and their attitudes towards PEP and immunisation at a regional hospital. Recommendations based on the findings will be made to improve adherence to the Act^2^ and the DOH guidelines.^3^

## Ethical considerations

Ethical approval was obtained from the Research and Ethics Committee at the University of KwaZulu-Natal. Permission to conduct the study was obtained from the hospital manager, the ethics committee at the hospital and the KwaZulu-Natal Department of Health.

Informed consent was obtained from all those who participated in the study. Patient information leaflets and consent forms were given to all participants in the study.

Data was protected by storing it in a lock-up safe that was only accessible to the researcher and the occupational health unit manager.

## Methods

### Design and procedure

A retrospective review of the records at the OHC of all doctors who sustained a sharp or mucosal injury between June 2010 and May 2011 was done. During the study it was noted that laboratory staff did not use the OHC as they were no longer employed directly by the DOH. All HCWs who have been exposed to blood or blood products presenting at the OHC are routinely tested for HBV and HIV antibodies as part of the PEP protocol at the hospital. Records were analysed retrospectively to determine if the HCW had immunity against hepatitis B at the time of the injury and whether or not they had been managed according to the DOH guidelines.^3^

In addition to the record review all laboratory personnel and senior doctors were asked to complete a self-administered questionnaire. Senior doctors were targeted as they presented a more stable doctor population; they are often asked by colleagues to explain the correct procedure following a NSI or other accidental exposure and are expected to set an example to junior colleagues. Over and above this, a brief review of the records at the OHC suggested that this group of HCWs generally did not attend the OHC for HBV immunisation or booster immunisation.

The questionnaire asked about previous sharps injury or mucosal exposure to blood or blood products and whether the HIV and the HBV status of the source patient had been checked at the time of injury. It aimed to discover whether or not the HCW was aware of his or her HBV immune status and assessed their knowledge and attitude regarding HBV infection.

### Setting

The study was conducted at a combined district and regional hospital in KwaZulu-Natal, South Africa. The hospital has 900 inpatient beds and serves up to 30 000 outpatients per month. The prevalence of HIV infection is very high, more than 60% of medical admissions being due to HIV related disease (personal communication by Dr Wilson, Head of the Department of Medicine). Although figures for HBV infection at the hospital are not known, SA has a high prevalence of HBV infection, more than 75% of adults having serological evidence of previous hepatitis exposure, and carrier rates between 10% – 25%.^6, 14^ The hospital employs 150 doctors of whom 100 are considered to be fulltime senior doctors, and 21 laboratory personnel.

### Materials

The study population included all senior doctors (medical officers, registrars and specialists) and laboratory personnel employed by the hospital. Doctors employed in all departments (anaesthetics, internal medicine, obstetrics and gynaecology, orthopaedics, paediatrics and surgery) were targeted.

### Analysing

The data were captured onto Microsoft Excel Spread sheets and imported into the Microsoft Excel program, version 2.22 Excel12+, for analysis.

## Results

Results were obtained from two sources, namely OHC records and the questionnaire.

Records from the occupational health clinic register showed that 67 doctors had reported a percutaneous or mucosal injury between June 2010 and June 2011. There were no records from laboratory personnel as they no longer access care at the OHC. Twernty-three of those who had sustained an occupational injury were men and 44 were women. The average number of years of service was 5 (range 1–8 years). The largest numbers of those with needle stick injuries were working in the department of surgery. This was followed by paediatrics and the least number of injuries were reported in the department of obstetrics and gynaecology (see Table 2).

**TABLE 2:** Status of percutaneous or mucosal injuries by discipline.

Injuries	*n*	%
Anaesthetic	9	13
Internal medicine	7	10
O & G	6	9
Ortho	7	10
Paeds	18	28
Surg	20	30

*n*, number of injuries.

At the time of the reported injury 39% of the injured doctors had had a negative Hep B surface antibody titre, indicating no immunity against HBV infection (see Table 3). Occupational health records revealed that only 5 of these HCWs had received the hepatitis B Immunoglobulin.

**TABLE 3:** Hepatitis B surface antibody status of injured health care worker.

Hepatitis B Antibody Status	*n*	%
Negative	26	39
Positive	33	49
Unknown (records not found)	8	22

*n*, number of workers.

Ninety-nine health care workers (78 out of 100 doctors and 12 out of 21 laboratory personnel) completed the questionnaire, giving an overall response rate of 74 percent. The average years of service were eight years.

In response to the questionnaire, 51 doctors reported at least one percutaneous or mucosal injury during their career. The HIV status of the source patient had been checked in 73% (37 out of 51) of the injuries whilest the HBV status of the source patient had been checked in only 4% (2 out of 51) cases.

Fifty-six percent of HCWs were not aware of their immune status. Furthermore only 22% had received a booster within 5 years of their last immunisation and 78% had received their booster after five years or not at all. Fifty-four percent of participants were not aware that the HBV booster should be administed every 5 years (see Table 4).

**TABLE 4:** Hazard for health care workers knowledge on frequency of administering Hepatitis B virus booster.

Knowledge of booster frequency	*n*	%
Yearly	13	15
Every 5 years	41	46
Every 10 years	5	5
I don't know	31	34

*n*, number of workers.

Assessment of the attitude of the participants regarding HBV revealed that 90% of the participants agreed that HBV PEP was as important as HIV PEP. Eighty-four percent of the participants also believed that it was important to check their HBV immune status at the time of an injury.

Their knowledge regarding HBV was weak. Only 33% of the participants knew the true risk of HBV transmission whilst 67% underestimated the risk.

Fifty-seven percent of staff correctly indicated that HBV infection was most easily transmitted after a needle stick injury. Thirty-five percent of staff incorrectly thought that HIV was more easily transmitted after an NSI than HBV.

## Discussion

Occupationally acquired HBV infection is a health hazard to HCWs who are not appropriately immunised. Despite the availability of the HBV vaccine this study showed that up to 61% of the doctors (39% known and 22% unknown) were not immune to HBV at the time of an exposure to potentially infected blood or body fluids. A statistically significant improvement is noted when these results are compared to those of a study by Vardas et al.^14^ in Johannesburg which showed that only 30.6% of HCWs were immune to HBV infection.

PEP against HBV infection is accessible in the form of the hepatitis B immunoglobulin. An injured HCW qualifies for this if he or she has not been immunised previously and the source patient is infected. Fifty-seven percent of doctors reported at least one sharp injury or mucosal exposure during their employment at the hospital, but only two of the source patients’ hepatitis B status had been checked. In 96% of cases their HBV status had not been checked. This is worse than the findings of De Villiers^8^ in Bloemfontein in 2007 which showed that the HBV status of 67.5% of patients remained unknown following occupational exposure by a HCW. In comparison the HIV status of the source patient is often known or actively sought and is considered the most important issue for the injured HCW, despite the fact that the risk of contracting HBV infection is 100 times higher than that of contracting HIV infection following a needle stick injury. Thirty-five percent of the participants incorrectly believed that HIV was more easily transmitted than HBV and this could explain why the HBV status of the source patient is not actively tested.

Although occupationally acquired HBV infection is preventable by vaccination, 56% of the participants were not aware of their immune status and 54% were not aware that the HBV booster dose should be administered every five years. After immunisation with the vaccine series it is vital to have an immunity test performed to determine if the HCW is protected against HBV. If the HCW does not develop an antibody response a second vaccine series needs to be given and the HCW rechecked to determine whether he or she is a responder or a non-responder.^13^ Every HCW should be aware of their immune status so that Hep B PEP can be managed appropriately. Human hepatitis B immunoglobulin is available in the public sector. Hepatitis B immunoglobulin should be administered within 48 hours to those HCWs who are not immune; however, it can be given up to seven days after exposure. At the hospital where the study was conducted, and in many other public sector hospitals in South Africa, it unfortunately takes more than seven days to obtain the results of the HepBsAb titre. This may limit the opportunity to access PEP. If the HCW in this hospital is unaware of his or her immune status, however, hepatitis B immunoglobulin must be given. In this study the immunoglobulin had been administered to only five HCWs between June 2010 and May 2011. This is a great concern as twenty-six doctors had not been immune at the time of the injury and as all should have been given the hepatitis B immunoglobulin in line with current policy. A further concern is that in 50% of injuries doctors had not returned to review their baseline blood results, including their HepBsAb titre, thus missing the opportunity to receive appropriate follow-up and immunisation.

More than 80% of participants acknowledged that HBV infection was important, yet 56% were not aware of their immune status. There may be a number of reasons for this, including the low profile of HBV infection, the pervasive nature of HIV infection and the emphasis that has been placed on HIV PEP. It may also be because 80% of the participants thought that the Hepatitis B immunisation programme at the hospital was neither well organised nor accessible.

These findings are comparable with a study carried out by Maha Talaat in Egypt which noted that only 15.8% of their HCWs completed the three doses of the hepatitis B vaccine.^17^

The majority of the injuries were reported by surgeons and by paediatricians. A study by Kent and Sepkowitz in 2004 showed that in prevaccine surveys the incidence of HBV infection was more than ten times higher in surgeons and laboratory workers exposed to blood and blood products than amongst other categories of HCWs.^18^ The least number of injuries were reported by doctors working in Obstetrics and Gynaecology. This finding was surprising as Obstetrics is a hands-on procedural discipline where needle stick injuries and mucosal exposure might be expected to occur.

Laboratory personnel have been employed by the National Health Laboratory Service since 2009 and they no longer access the service at the OHC. Results on exposure to blood or blood products were therefore not available. 80% were aware of their HBV immune status, but the majority had poor knowledge regarding the risk of acquiring HBV. The majority also believed that HIV was more easily transmitted than HBV after an injury. No injuries were reported in this group.

South Africa has a high prevalence of HBV infection, more than 75% of adults having serological evidence of previous hepatitis exposure and carrier rates between 10% – 25%.^14^ HCWs are therefore susceptible to and at risk of acquiring this preventable disease. The onus is on HCWs to take responsibility for their own health. The National DOH can assist by introducing legislation similar to legislation in Britain,^19^ requiring all HCWs to submit proof of their HBV immunity prior to being employed.

## Limitations of the study

This was a small sample done in only one hospital and the results may not be reflective of all HCWs in South Africa. Not all injuries are reported to the OHC and this may have led to information bias. The questionnaire required information from previous NSIs or mucosal exposure to blood or blood products and these requests may have introduced some informational bias. However, these events are often traumatic and HCWs would be expected to remember details of such events.

## Conclusion

Poor compliance of HCWs to hepatitis B vaccination and PEP is a grave concern as HBV infection is associated with serious public and personal health consequences. It is evident from the results above that doctors are not appropriately protected against a preventable occupational disease. This may be due to obvious challenges in institutions, such as a lack of structured health promotion programmes on an on-going basis. Poor budget allocation, lack of management support, poor health and safety standards in general and a discordant access to occupational health care services in most public hospitals in South Africa may also partly explain the phenomenon. In-service training is needed to ensure that doctors are appropriately informed on the safety and efficacy of hepatitis B vaccination and PEP. Legislation requiring proof of immunity before confirmation of employment could substantially reduce any risk of HBV infection to HCWs.
